# The Irritation of Rubber Plates

**Published:** 1873-12

**Authors:** 


*The Irritation of Rubber Plates.*—Dr. C. C. Knowles professes to remedy irritation from rubber plates by employing black vulcanite instead of red, and also to dispense with the " central oavity." He says :

" I have met many cases of irritation of the mucous membrane, especially in the fauces caused by the irritation of the rubber plate. I have substituted black vulcanite, and the irritation entirely ceased. I have been in the habit of using it on account of its strength, especially in partial sets, where we demand a greater strength in proportion to the surface covered. It receives a most beautiful polish, and its texture shows it to be more dense.

" The central cavity may be useful in temporary sets ; but after the absorption of the gums has taken place, I think the plate can be better secured in the mouth by a bead around the circumference of the plate, so that it shall perfectly embrace the parts, taking care that it does not come up too high, and that the beading is not so deep as to cause a stoppage of the circulation. Properly placed, it will allow the movement necessary for mastication without displacement. I find such plates give satisfaction and less irritation to the mouth. I trim it so that the beading shall fall one or two lines within the extent of the plate, as it covers the gum, and within a line or two of its terminus. The terminal edge as it comes against the soft tissues, should not be too sharp, but should be rounded out, especially over the molar process, as there is where irritation is liable to occur."

